# Clinical Implications of the Molecular and Genomic Landscape of Upper Tract Urothelial Carcinoma

**DOI:** 10.1007/s11934-024-01245-1

**Published:** 2024-10-08

**Authors:** Salvador Jaime-Casas, Abhishek Tripathi, Sumanta K. Pal, Wesley Yip

**Affiliations:** 1 Department of Medical Oncology & Experimental Therapeutics, City of Hope Comprehensive Cancer Center, 1500 East Duarte Road, Duarte, CA 91010 USA; 2 Division of Urology and Urologic Oncology, City of Hope Comprehensive Cancer Center, 1500 East Duarte Road, Duarte, CA 91010 USA

**Keywords:** Genomics, Proteomics, Molecular, Urothelial

## Abstract

**Purpose of Review:**

Upper tract urothelial carcinoma (UTUC) is an aggressive entity with treatment strategies mirroring bladder cancer. Genomic and molecular profiling allows for a better characterization of this disease and allows for patient-tailored approaches. We aim to describe the genomic and molecular implications of this disease.

**Recent Findings:**

Technological advances have the potential for early diagnosis and precise molecular analysis in patients with UTUC. Genomic profile clustering, specific mRNA signatures, and pathway-specific protein abundance tools have oncologic and clinical implications. We describe their utility in the context of this disease.

**Summary:**

In the era of precision medicine, designing clinical trials that explore the diagnostic and prognostic implications of biomolecular signatures in the context of UTUC is of utmost importance. Promising advances in this arena provide tools for physicians to avoid overtreatment in this patient population.

## Introduction

Urothelial carcinoma (UC) is the sixth most common malignancy worldwide and can occur anywhere along the urinary tract. Localization in the bladder occurs in 90–95% of cases, while growth in the upper tract is relatively uncommon, representing only 5–10% of cases [1]. The incidence of the latter has risen in the past few years as advances in diagnostic technology improve the detection rate in patients [2]. At diagnosis, approximately 50% of patients have non-muscle invasive disease, while the other 50% present with muscle-invasive or non-organ confined disease. Up to 25% of patients will present with metastatic disease [2]. Although the clinical spectrum between urothelial carcinoma of the bladder (UCB) and upper tract urothelial carcinoma (UTUC) has some overlap, evidence in the molecular and genomic arenas supports the concept of them being separate entities. For example, a genomic analysis performed by Memorial Sloan Kettering Cancer Center revealed that *FGFR3* and *HRAS* alterations are more prevalent in UTUC. At the same time, *TP53*, *ERBB2*, and *RB1* mutations are more predominant in UCB [3]. These differences may explain the distinct clinical behavior between both entities and could pertain to the more aggressive disease course seen in UTUC [3–5]. This is especially true for Asian patients, as a recent evaluation of the Surveillance Epidemiology and End Results Database from 2004 to 2016 found that among patients with non-metastatic UTUC who underwent radical nephroureterectomy (RNU), Asian patients exhibited the highest rate of more than two positive lymph nodes relative to other races [6]. Similarly, after propensity score matching and multivariable-adjusted analysis, Asian race independently predicted cancer-specific mortality relative to Caucasians (HR 1.29, *P* <0.01) [6]. Lynch syndrome, an autosomal dominant hereditary syndrome characterized by alterations in mismatch repair genes (*MLH1, MSH2, MSH6, or PMS2*), is also strongly associated with the development of UTUC, particularly in younger patients [7].

The genomic and molecular characterization of UTUC is crucial to advancing diagnostic and therapeutic efforts forward. Most current clinical trial designs and research frameworks focus on UCB. However, given the significantly distinct molecular profiles between UCB and UTUC, perhaps they should be evaluated separately. For this reason, this review will provide an overview of the genomic and molecular landscape of UTUC, as well as the clinical implications this entails.

## Molecular and Genomic Clusters

The evaluation of UTUC consists of both non-invasive and invasive modalities. However, unlike their bladder counterparts, these modalities pose the challenge of having decreased sensitivity to evaluate muscle-invasive disease, as pathology specimens can be limited due to the high risk of ureteral perforation [8]. This is quite problematic, as physicians can initially struggle to characterize disease stages accurately, and patients are vulnerable to suffering from disease under-staging or overtreatment [8]. One way to potentially overcome this is by using integrative prognostic models [9]. Petros et al. designed a preoperative predictive model to identify high-risk non-organ confined UTUC. This model integrates tumor grade, tumor architecture, clinical stage, and hemoglobin, demonstrating an accuracy of 82% [10]. In the postoperative setting, the model proposed by Yates et al. aims to predict cancer-specific survival after RNU based on tumor stage, lymph node involvement, tumor grade, patient age, and tumor location. Accuracy after internal and external validation was 78% and 71.8%, respectively [11, 12]. There is also an effort to improve upon traditional diagnostic tools by utilizing genomic sequencing. Katims et al. evaluated 48 patients with UTUC and performed next-generation sequencing on their urine cytology samples to evaluate the genomic concordance of identified mutations with the primary tumor tissue. In 94.4% of patients (*n* = 34/36), cytology showed at least one shared mutation with the tissue sample. The overall concordance rate for their cohort was 55%. Similarly, 30.6% of patients (*n* = 11/36) elicited 100% concordance between cytology and tumor tissue [13]. In patients who end up undergoing RNU, final pathology reveals high-grade disease in 70% and muscle-invasive disease in 60% of cases. These findings entail a poor prognosis, with a 5-year cancer-specific survival of less than 50% for T2/T3 disease and 10% for T4 disease [14].

Grahn et al. described the landscape of genomic alterations in patients with UTUC who underwent nephroureterectomy. They found that patients who died from UTUC had specific *TP53* and *HRAS* mutations. Likewise, they found that *FGFR3* mutation is associated with improved prognosis in patients with Ta grade 1 disease, as no patient in this group died regardless of primary tumor size or multifocality [15, 16]. Granted, this clinical stage has a better prognosis than advanced ones. Fujii et al. profiled the molecular and genomic landscape of 199 UTUC samples, clustering them into five unique mutational subtypes. These were categorized as *TP53/MDM2, RAS, FGFR3*, triple-negative (no alterations in *TP53/MDM2, FGFR3*, and *RAS* genes), and hypermutated (based on the degree of positive mutational signatures of the genes). After evaluating oncologic outcomes, the hypermutated and *FGFR3*-mutated cohorts had the best disease-specific survival among all groups. The triple-negative and the *TP53/MDM2*-mutated groups showed worse disease-specific survival. Although clinical and histological characteristics are strongly implicated in survival outcomes, their multivariate analysis showed that genomic factors explained 40% of the hazard [4]. Similarly, clustering of UTUC cases may provide insight into future therapeutic implications, as authors also described that the hypermutated and *TP53/MDM2*-mutated cohorts expressed a high tumoral-mutational burden and increased expression of immune checkpoint inhibitor molecules, making them attractive prospects for immune-checkpoint inhibitor-based therapies [4]. In a similar study, 100 UTUC tumors from patients who underwent RNU were grouped into five clusters according to their molecular profiles. Clusters one and two were associated with pT3/T4 disease and worse disease-free (DFS) and overall survival (OS). Cluster five was associated with pTa/T1 disease and better DFS and OS. After genomic analysis, clusters one and two showed increased inflammation signatures composed of TNF-α, IL-6 JAK/STAT3 signaling, IL-2, and PDL-1 [17]. Both studies highlight the implications of molecular and genomic clustering on clinical outcomes in patients with UTUC.

Moreover, this technique provides insight into therapeutic interventions that could benefit patients. For example, Van Allen et al. identified *ERCC2*, a nucleotide excision repair gene, to be preferentially expressed in patients with muscle-invasive bladder cancer who were cisplatin responders in the neoadjuvant setting [18]. Similarly, Hirotsu et al. sequenced genomic alterations in 19 patients with UCB. They found that of 142 mutations, patients harboring *ERCC2*-mutated signatures showed improved response to platinum-based chemotherapy, as evidenced by the decrease in size of the primary tumor and lymph node metastases, as well as decreased expression of tumor-derived DNA in urine samples [19]. However, the therapeutic implication that *ERCC2*-mutated UTUC tumors have is yet to be determined [20]. Since genomic information was obtained from resected tumor tissue, it provides an opportunity to retroactively evaluate tumor characteristics more commonly implicated in treatment response and aggressiveness. This could help determine, perhaps from biopsy specimens, which patients are candidates for systemic therapy and kidney-sparing approaches.

## Circulating Tumor DNA

### Plasma-derived

Circulating tumor DNA (ctDNA) is tumor-shed cell DNA in a patient's plasma. Identifying tumor-specific alterations in this biomarker is a growing area of focus with diagnostic and prognostic applications. Detectable levels of ctDNA in UCB can be found in 35% of patients with localized disease and up to 80% of patients with metastatic disease [21–23]. Likewise, increased levels of ctDNA in this patient population have been implicated with a more aggressive disease course, as well as an increased risk of recurrence and short-term metastasis [22]. In the setting of UTUC, Huelster et al. recently evaluated the usefulness of plasma ctDNA in identifying muscle-invasive and non-organ-confined disease. They conducted a prospective analysis of 30 patients with chemotherapy naïve, high-risk disease, undergoing surgical extirpation. Utilizing ctDNA to detect molecular panel-based alterations yielded a sensitivity of 71% and a specificity of 94% in predicting muscle-invasive/non-organ-confined disease [24]. Of note, six patients (20%) had low-grade disease, which could have hindered the performance of their model. Current results suggest that ctDNA could potentially help characterize patients with presumably localized disease who are not actually organ-confined or have a higher risk for recurrence or progression and may benefit from neoadjuvant systemic therapy [24]. Mu et al. recently performed a pilot study investigating if targeted genetic analysis of plasma ctDNA could identify tumor-specific gene variants. ctDNA-related alterations were seen in four of six total patients, particularly in those with ≥ grade 2 disease and those with > 300m2 tumors in size. However, their smaller sample size is a limitation of this study [25].

### Urine-derived

Tamura et al. evaluated the utility of ctDNA as a biomarker for predicting disease recurrence in 23 patients undergoing nephroureterectomy and followed for two years. Urine and blood samples were collected at different points in time. All patients with intravesical recurrence were ctDNA positive in urine, with positivity becoming apparent 60 days before cystoscopy detection. Likewise, ctDNA became positive in plasma when metastasis was documented for patients with metastatic disease. Although some limitations of this study are the low number of metastatic patients (*n* = 2) assessed and the single-center design, it does show precedent for the utility of this non-invasive biomarker [26]. Fujii et al. investigated the performance of urinary sediment-derived DNA in detecting a pool of 30 genes commonly mutated in UTUC. They found that sequencing of urinary sediment-derived DNA had 82.2% sensitivity and 100% specificity for cancer detection, outperforming urinary cytology (32.9% sensitivity and 88.9% specificity). Interestingly, 8 out of 13 false-negative samples derived from patients with hydronephrosis. Specifically, when authors analyzed a patient with severe hydronephrosis they found that the urine sample collected upstream from the obstruction successfully detected all mutations found in the primary tumor. This highlights hydronephrosis as a potential confounder for cfDNA interpretation in urine samples [4]. Patients with UTUC are particularly prone to misclassification, as urine cytology has poor sensitivity for low-grade disease, and ureteroscopic evaluation is an invasive procedure [27]. ctDNA has good diagnostic performance, and its low-invasive nature makes it an attractive modality.

## Proteomics

Plasma proteins are an abundant component of plasma and oversee multiple cellular processes involved in growth, signaling, transportation, and response to tissue damage [28]. With advanced proteomics technology, protein-specific signatures and their association with various pathologic conditions have been identified [29]. Qu et al. analyzed plasma and urine proteomic profiles of patients with UTUC and healthy controls at different points in time. They found that most proteins associated with UTUC were kidney-tissue-specific, hypothesized to be because of tumor growth and subsequent cell death with the release of intracellular proteins. Specifically, most were found to be involved in fatty-acid metabolism pathways. In contrast, the proteomic profile of healthy controls was mainly associated with cellular sodium-ion homeostasis. Upon further stratification, patients with muscle-invasive disease showed a proteomic profile involved in acute-phase inflammatory response and fatty-acid degradation. Patients with non-muscle invasive disease showed a proteomic profile associated with sulfur metabolism and deubiquitination pathways. Ultimately, authors were able to cluster four fibrinogen-related proteins (SAA1, CRP, SAA2, and ITGB2) that correlated with worse OS and progression-free survival (PFS) [30]. In a similar study, Lu et al. performed gel electrophoresis of total protein samples in 19 patients with UTUC and 20 healthy controls. In their results, increased expression of annexin A2, annexin A3, and calreticulin in urine samples was predominantly expressed in UTUC patients, suggesting their potential as a urine biomarker in this patient population. However, the diagnostic performance of these markers is yet to be evaluated [31]. Li et al. performed serum analysis to identify which metabolic markers were predominantly expressed in 39 UTUC patients compared to 34 healthy controls. Serum metabolomic analysis revealed that the UTUC population expressed high levels of lactate and creatinine and an upward trend in serum polyunsaturated fatty acids and 3,7-dimethyluric acid [32]. Proteomics can group specific protein signatures involved in various cellular processes with disease stages and behavior. Identifying specific metabolic pathways implicated in tumor-abundant and tumor-free environments allows for a better understanding of disease behavior.

## Micro-RNA Signatures

Micro-RNA (miRNA) corresponds to a non-coding sequence of RNA that regulates protein synthesis by binding transcript components and overseeing messenger RNA (mRNA) degradation or translation [33, 34]. This regulatory mechanism has been involved in cancer biology, and the evaluation of specific miRNA signatures is increasingly considered to play a role in UTUC [35]. Izquierdo et al. analyzed miRNA expression in tumor samples of patients with progressing and non-progressing UTUC. They found that miRNA-31 and miRNA-149 signatures were independent predictors of tumor progression. Likewise, miRNA-149 expression was also implicated in predicting cancer-specific survival [36]. There is also literature supporting the concept of miRNA-149 dysregulation being expressed in other genitourinary neoplasms like clear-cell renal cell carcinoma and prostate cancer [36]. However, validating these miRNA signatures in clinical practice warrants more extensive research. Browne et al. analyzed 157 radical nephroureterectomy specimens from two institutions to determine which miRNA signatures were associated with disease-grade characterization. High-grade UTUC showed increased expression of miRNA-29b-2-5p, miRNA-18a-5p, miRNA-223-3p, and miRNA-199a-5p. After analyzing their diagnostic performance, these signatures generated an area under the curve of 0.86. Similarly, muscle-invasive disease showed increased expression of miRNA-10b-5p, miRNA-26a-5p-5p, miRNA-31-5p, and miRNA-146b-5p, generating an area-under-the-curve of 0.90 [37]. This study was one of the first to show the diagnostic performance of specific miRNA signatures in patients with UTUC, particularly in discerning high-grade and muscle-invasive disease status. Montalbo et al. assessed the predictive value of miRNA signatures in patients with UTUC. In their analysis, increased expression of miRNA-151b was associated with decreased tumor progression and better cancer-specific survival [38]. Analysis of miRNA signatures is an area of research that has increased in popularity in recent years. Its low-invasive nature and relatively good stability in tissue and serum samples make it an appealing landscape compared to other non-invasive techniques [39].

## Lynch Syndrome and UTUC

Lynch syndrome (LS), also known as hereditary non-polyposis colorectal cancer (HNPCC), was initially described by Lynch et al. in 1966 [40]. This condition is caused by a germline mutation in one of four mismatch repair (MMR) genes: *MLH1, MSH2, MSH6,* or *PMS2*. Mutations involving the *EPCAM* gene also result in MSH2 inactivation and thus have been described as another genetic driver [41]. Unlike sporadic UTUC, LS-associated UTUC has a higher female prevalence, a younger age of onset, and a higher predisposition to bilaterality [42].

Patients with LS have a lifetime risk of developing colorectal cancer of 70%, endometrial cancer of 50%, and UTUC of 20%. Although UTUC is the most likely urological malignancy to develop, with a 14-fold increased risk compared to the general population, UCB, prostate, and testicular cancer are underrecognized malignancies also implicated in this syndrome [43, 44]. Because of this reason, precision medicine is necessary when encountering patients with confirmed or suspected LS. Currently, the American Urological Association and the European Association of Urology recommend performing systematic screening when facing a patient with UTUC and age of onset < 60 years, a personal history of LS, or one or more first-degree relatives with Lynch-spectrum malignancies [45, 46]. However, treatment guidelines do not distinguish between LS- and sporadic-UTUC, as both consider risk stratification and favor kidney-sparing approaches when possible [47–49]. Identifying MMR-deficient genes in patients with LS can work as a therapeutic target. Some studies have evaluated the performance of immune checkpoint inhibitors (ICI) in this patient population. However, due to the low prevalence of LS-related UTUC, most of the benefits are extrapolated from studies evaluating LS patients with non-colorectal malignancies. Raj et al. identified two patients with LS-associated adrenocortical carcinoma receiving pembrolizumab. Patients one and two responded to treatment as early as 5.5 and 9 weeks of therapy, respectively [50]. Likewise, in a preliminary study by Doudt et al. ten patients with LS-associated UTUC treated with ICI were evaluated. Six patients had metastatic disease, of which four were progression-free at 24 months. Similarly, four patients had localized disease, of which three showed complete pathological response [51]. Although this study is limited by its population size, it denotes the clinical benefit of ICI in LS-associated UTUC.

Genomic characterization has pointed out the specific implications that MMR mutations have on the development of other related conditions. For example, *MSH2* has been singled out as a potential driver for the development of UCB when compared to other MMR alterations [43]. Whole exome genomic analysis allows individualized risk stratification depending on the genomic profile of patients. A more widely available resource is immunohistochemistry (IHC), which can identify altered LS-associated proteins and help screen patients who require further confirmatory genetic testing. It is of utmost importance that general urologists and non-urologic primary care physicians screen patients with UTUC with IHC for LS-related pathogenic variants. Lowering the referral threshold to urologic oncologists and comprehensive cancer centers for this group of patients could help identify the real prevalence of this condition and allow for early detection of hereditary neoplasms in patients and family members.

## Clinical Trials: Molecular and Genomic Study Designs

Clinical trials designed to explore different diagnostic and therapeutic interventions in the context of UTUC represent a small fraction of available genitourinary clinical trials. Most studies include unselected patient populations with UTUC and do not consider specific molecular or genomic profiles. In this regard, the POUT trial evaluated cisplatin or carboplatin in combination with gemcitabine in 261 patients with muscle-invasive or lymph node-positive, nonmetastatic UTUC who were randomly assigned after undergoing radical nephroureterectomy to platinum-based chemotherapy (*n* = 132) or surveillance (*n* = 129). At 5-year follow-up, DFS was 62% in the chemotherapy arm versus 45% in the surveillance arm (HR 0.55; *P* = 0.001). Also, OS was 66% in the chemotherapy arm versus 57% in the surveillance arm (HR = 0.68; *P* = 0.049). Although both chemotherapy regimens showed improved benefits compared to surveillance alone, the cisplatin-based group showed superior DFS (HR 0.53; 95% CI 0.33, 0.86;) and OS (HR 0.57, 95% CI 0.33, 0.97;) [52]. The PURE-02 study evaluated pembrolizumab in the neoadjuvant setting in ten patients with high-risk UTUC (defined by the presence of either high-grade disease on urinary cytology and/or biopsy, multifocal disease, ≥ 2 cm tumor mass, or hydronephrosis) (N0M0). In their results, one patient achieved a complete clinical response, two had disease progression, and seven were non-responders. Not only were the results not promising, but the authors also highlighted the biomarker-unselected nature of their population, indicating that treatment response could be dubious in unselected patients [53]. The ECOG-ACRIN EA8192 is a phase II/III trial evaluating chemotherapy alone or in combination with durvalumab in patients with high-grade UTUC prior to nephroureterectomy, making it one of the few to actively evaluate combined therapy in this disease setting.

Of interest are two trials evaluating FGFR3-targeting drugs in patients with UTUC. The PROOF-302 (NCT04197986) trial investigated the efficacy and safety of adjuvant infigratinib (a selective FGFR1-3) vs placebo in patients with high-risk invasive urothelial carcinoma. Their cohort included 188 patients with FGFR1-4 gene alterations (102 with UTUC, 85 with muscle-invasive bladder cancer, and 1 with unknown origin). However, the trial was stopped early by the sponsor due to the low prevalence of FGFR3 alterations, found only in 19% of the screened population. Despite this, the genomic analysis provided insight into the prevalence and pattern of the FGFR3 mutation profile, showing that FGFR3 was the predominant mutation in UTUC and muscle-invasive bladder cancer (67% and 55%, respectively). Moreover, most FGFR3 mutations in UTUC were single-nucleotide variations (56%), while for muscle-invasive bladder cancer, most were amplifications (60%) [54]. Similarly, a recent phase 1b trial (NCT04228042) evaluated infigratinib as neoadjuvant treatment in 14 patients with localized UTUC undergoing ureteroscopy or nephroureterectomy/ureterectomy. Results showed good tolerability and a promising response profile, with 6 in 9 patients with FGFR3 alterations eliciting response to treatment. Of note, responders had a median tumor size reduction of 67% [55]. Both studies highlight the potential therapeutic benefit of biomarker-driven and FGFR3-targeting clinical trials. Considering recent advances in molecular and genomic characterization of UTUC, developing clinical trials using biomarker-driven criteria should be standard for interventional research.

We highlight four clinical trials with molecular-tailored designs in UTUC. The AnchorDx BC006 study (NCT04948528) evaluates DNA methylation/somatic mutation in patients with UTUC. The UTUC-ADJ-MDR trial (NCT05595408) evaluates circulating and urine tumor DNA to detect minimal residual disease and early diagnosis of recurrence in patients with UTUC. The CIRCE (NCT06116396) trial evaluates the prognostic role of circulating tumor cells in patients with UTUC and UCB. The LS-URO trial (NCT06218433) evaluates the use of urine tumor DNA in patients with LS, irrespective of primary tumor location in the urinary tract. Although promising examples, there is still a lack of interventional clinical trials with selected population designs. Since the genomic and molecular landscape is being addressed, centers should be motivated to develop interventional studies around selected molecular patient profiles. A detailed description of these clinical trials can be found in Table 1.

**Table 1:** List of clinical trials with molecular/genomic characterization design in UTUC

Design	Drug/Intervention	Trial / NCT	Patient population	Outcomes to evaluate
Prospective, multi-institutional, observational cohort	DNA methylation/somatic mutation test	AnchorDx BC006 / NCT04948528	Patients with suspected UTUC	Sensitivity and specificity
Prospective, observational cohort	ctDNA and utDNA to detect MRD	UTUC-ADJ-MDR / NCT05595408	Patients with locally advanced UTUC who underwent radical nephroureterectomy	2-year recurrence free survival
Case control, observational	CTC and UTC in the prognosis and therapy of patients with UCB and UTUC	CIRCE / NCT06116396	Cohort 1: urothelial neoplasia Cohort 2: age-matched, neoplasia-free individuals	Prognostic role
Single group, interventional	Urothelial cancer screening using urine tumor DNA test	LS-URO Study / NCT06218433	Patients with Lynch syndrome	Sensitivity and specificity

Registered clinical trials with a molecular/genomic approach to UTUC

*ctDNA*, Circulating Tumor DNA; *CTC*, Circulating Tumor Cells; *MRD*, Minimal Residual Disease; *UTUC*, Upper Tract Urothelial Carcinoma; *UTC*, Urinary Tumor Cells; *utDNA*, Urine Tumor DNA; *UCB*, Urothelial Carcinoma of the Bladder

## Future Perspectives

Although advances in research focused on UTUC have been made, many techniques under evaluation are constantly showing their diagnostic and prognostic potential. As they evolve, the arsenal of precision-based tools for UTUC will expand significantly. Some of the notable techniques under development are:

### Nectin-4 as a Potential Therapeutic Target

Nectin-4 is a transmembrane protein involved in calcium-independent cellular adhesion expressed in approximately 60% of urothelial cancer cases [56]. This has led to the evaluation of enfortumab vedotin (EV), an anti-Nectin-4 antibody–drug conjugate, in multiple trials [57–59].

Calandrella et al. evaluated a cohort of 27 patients with UTUC undergoing RNU and found that Nectin-4 was expressed in 44% of their patient population [60]. In a similar analysis by Hashimoto et al., authors described a Nectin-4 expression of 65% [61]. Although these studies are not as robust as those evaluating this biomarker in bladder cancer, they show comparable prevalence and could hypothetically also be susceptible to therapeutic targeting with EV. The EV-302 study compared the efficacy of EV and pembrolizumab against platinum-based chemotherapy in patients with treatment-naïve locally advanced or metastatic urothelial carcinoma. Results showed improved progression-free survival (median 12.5 months vs 6.3 months; *P* <0.001) and overall survival (median 31.5 months vs 16.1 months; *P* <0.001) in the combination arm compared with chemotherapy. Although this study was not powered to show a benefit in patients with UTUC, 27% of their population had upper-tract disease as the primary disease site, thus showing the potential therapeutic benefit in this entity [62].

### 3D Microscopy

3D microscopy is a novel technique that allows for the three-dimensional visualization of samples. Samples are rendered under a light microscope, generating images that can achieve single-cell resolution. A clear advantage of 3D microscopy is that tumor microenvironment, including cellular and non-cellular components, can be analyzed simultaneously with high-precision software. Tanaka et al. evaluated this technique in 44 urothelial carcinoma samples, 40 of which were UCB and 4 were UTUC. They were able to describe the phenotypic landscape of microtumoral heterogeneity, and the ratio of various tissue-to-angiogenesis niches within the tumor [63]. Grahn et al. utilized this same technique to visualize two TaG1 and two T3G3 formalin-fixed paraffin-embedded UTUC samples. They quantified CD34 density, vessel radius, and sample heterogeneity. The CD34 density kurtosis and skewness were markedly elevated in high-grade disease compared to low-grade disease. Similarly, high-grade and low-grade tumor types differed distinctly in the parameters analyzed from normal urothelial tissue [64]. 3D microscopy software can be enriched with various phenotypic parameters, more precisely describing tumor microenvironment characteristics and disease staging.

### Extracellular Vesicles

Extracellular vesicles are cell-derived membrane structures that contain genetic material (mRNA), proteins (enzymes, growth factors, cytokines), or metabolites. They function through paracrine signaling of extracellular components. In cancer cells, extracellular vesicles containing growth factors play a crucial role in tumor cell accelerated growth, angiogenesis, and overall development [65]. Conversely, extracellular vesicles derived from mesenchymal/dendritic cells can promote anti-tumor effects in the tumor microenvironment. Enhancement and therapeutic targeting of the latter has been an area of focus. In a study by Eldh et al. exosomes from bladder tissue samples of sites close to or distant from the primary tumor were analyzed. Results showed that cancer-related proteins were abundant in exosomes regardless of the sampling site, suggesting that the entire microenvironment is filled with cancer-related exosomes even when the primary tumor has been locally resected or is in a distant location [66]. This highlights exosomes' potential to determine residual disease not visible under regular cystoscopy surveillance and the requirement for more aggressive treatment in patients with cancer-enriched microscopical niches [66]. In a similar study by Hiltbrunner et al. 13 patients with UCB were investigated using urine samples to determine if pro-carcinogenic exosomes could be detected in urine after histological staging. Vesicles were clustered according to bladder tissue with and without tumor contact or ureteral tissue without tumor contact at cystectomy. Although patients were tumor-free after cystectomy, urine derived from the bladder was enriched with carcinogenic-metabolite exosomes. Authors hypothesize this is the result of undetected or partly transformed cancer cells that continuously release carcinogenic exosomes and potentially promote recurrence [67]. Although both studies show promise in evaluating exosome profiles in urothelial carcinoma, upper tract-specific designs are warranted to understand its potential for disease surveillance and activity.

### Tumor Microenvironment Analysis

**Fig. 1:**
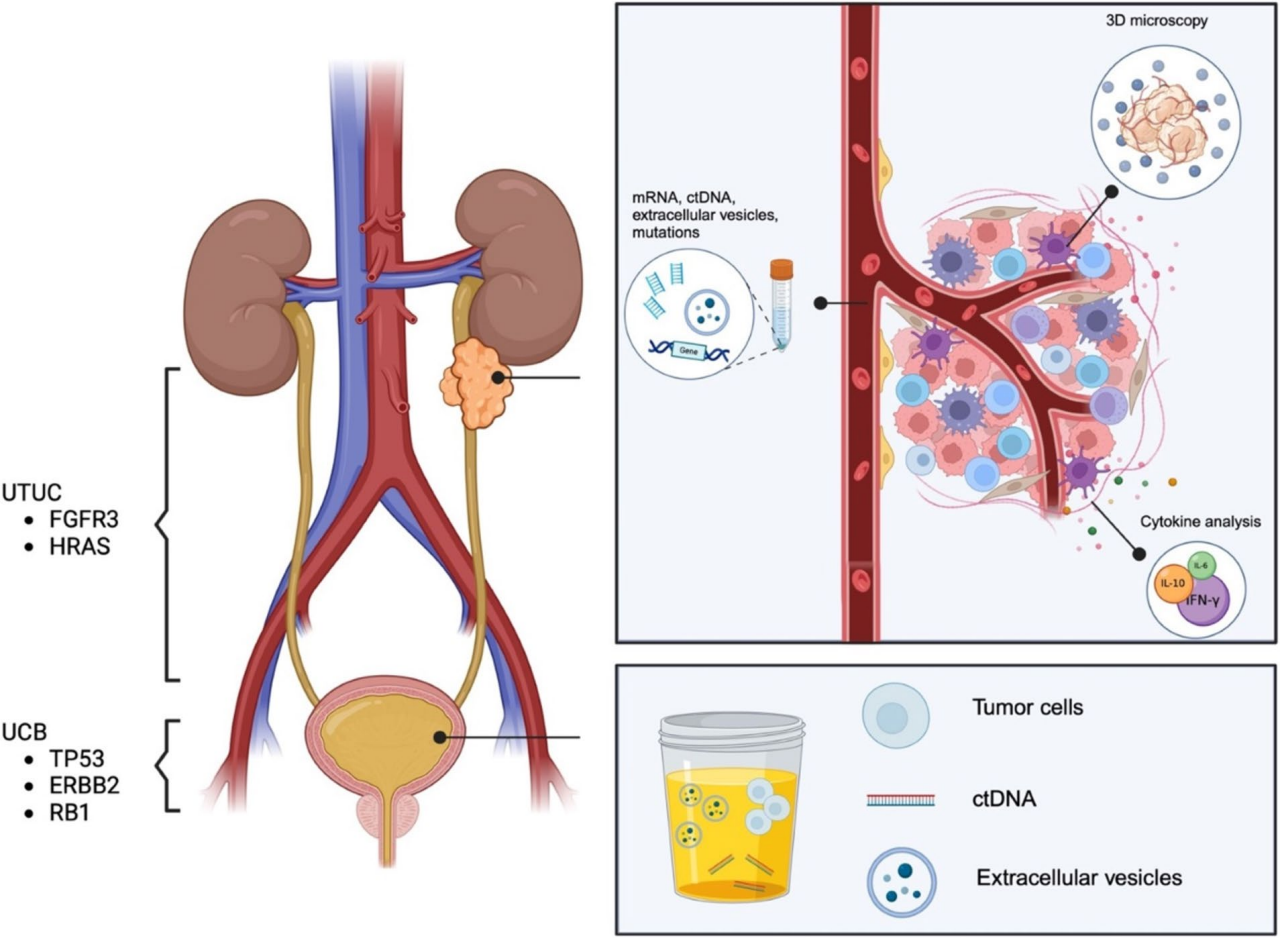
Landscape of emerging modalities in the characterization of UTUC. Genomic landscape of UTUC and UCB, as well as emerging diagnostic modalities for UTUC evaluation. ctDNA, circulating tumor DNA; UCB, urothelial carcinoma of the bladder; UTUC, upper tract urothelial carcinoma

Durable effects associated with systemic therapy involve promoting or inhibiting a particular immune system element. Achieving a durable and persistent response after systemic therapy, particularly immunotherapy, is correlated with a solid and sustainable effector cytotoxic response, mainly carried out by intratumoral CD4 + and CD8 + cells [68]. In the context of UCB, research suggests that an immune-rich tumor microenvironment is necessary and associated with a better response to immunotherapy. In a study by Deng et al. tumor samples from patients with UCB who had received pembrolizumab or atezolizumab/durvalumab were analyzed. Patients with more satisfactory responses had a higher clonal diversity and frequency of effector cells and stronger CD4 + gene signatures in their samples. Likewise, IHC analysis demonstrated that more CD8 + cells within UCB tissues were significantly associated with prolonged cancer-specific survival in patients receiving immune checkpoint-inhibitor therapy [69]. In the same study, sequencing tumor-infiltrating lymphocytes revealed that patients with higher clonal expansion had longer cancer-specific survival when compared to those with lower clonal expansion [69, 70]. This evidence supports the need for personalized medicine derived from microtumoral analysis. Although most published evidence is UCB-related, this works to set a precedent for feasibility and emphasizes the need to extend this area of research to patients with UTUC. This landscape of novel approaches and genomic underlining for UTUC is visualized in Fig. 1.

## Conclusions

Our understanding of the genomic and molecular implications driving UTUC has vastly expanded since it was initially described, from being considered part of the spectrum of UCB to becoming an independent entity with specific clusters of genomic and molecular signatures. Despite these advances, there is still much roadwork ahead to achieve precision-based interventions in the context of UTUC. The need to develop clinical trials and multi-center studies around this disease's molecular and genomic characterization is pressing. We take relief in the extensive, high-quality work researchers have done worldwide to provide evidence that fuels the development of patient-tailored strategies and guidelines.

## Key references

- Fujii Y, Sato Y, Suzuki H, et al. (2021) Molecular classification and diagnostics of upper urinary tract urothelial carcinoma. Cancer Cell 39:793–809.e8.
  - ○ This study underscores the importance of molecular diagnostics in enhancing the understanding and tailored treatment of patients with UTUC.
- Katims AB, Gaffney C, Firouzi S, et al. (2023) Feasibility and tissue concordance of genomic sequencing of urinary cytology in upper tract urothelial carcinoma. Urologic Oncology: Seminars and Original Investigations 41:433.e19-433.e24.
  - ○ This study highlights the performance of combining urine cytology, a traditional diagnostic tool, with next-generation sequencing in patients with UTUC.
- Huelster HL, Gould B, Schiftan EA, et al. (2024) Novel Use of Circulating Tumor DNA to Identify Muscle-invasive and Non–organ-confined Upper Tract Urothelial Carcinoma. Eur Urol 85:283–292.
  - ○ This study demonstrates the utility of plasma-derived circulating tumor DNA (ctDNA) in predicting high-risk and advanced disease.

## Data Availability

No datasets were generated or analysed during the current study.
